# Therapeutic Potential of *Adina rubella* Hance Stem and Picroside III as a Differentiation Inducer in AML Cells via Mitochondrial ROS Accumulation

**DOI:** 10.3390/ijms26031350

**Published:** 2025-02-05

**Authors:** Chan-Seong Kwon, Byeol-Eun Jeon, Ji-Eun Lee, Hyeon-Young Kim, Ryun-Young Kang, Keun-Hu Kim, Eun-Ju Lee, Ju-Yeon Jang, Tae-Jin Kim, Ho-Jin Shin, Sang-Woo Kim

**Affiliations:** 1 Department of Integrated Biological Science, Pusan National University, Busan 46241, Republic of Korea; ckstjd5091@naver.com (C.-S.K.); starsilver20@naver.com (B.-E.J.); dlwldms4535@naver.com (J.-E.L.); 2 Department of Molecular and Biopharmaceutical Sciences, Graduate School of Convergence Science and Technology, Seoul National University, Seoul 08826, Republic of Korea; hysnu0703@snu.ac.kr; 3 Department of Biological Sciences, Pusan National University, Busan 46241, Republic of Korea; coolsun1206@naver.com (R.-Y.K.); kkk030303@gmail.com (K.-H.K.); agju0108@gmail.com (E.-J.L.); jangjy05@pusan.ac.kr (J.-Y.J.); tjkim77@pusan.ac.kr (T.-J.K.); 4 Division of Hematology-Oncology, Department of Internal Medicine, Pusan National University Hospital, Busan 49241, Republic of Korea; hojinja@hanmail.net

**Keywords:** acute myeloid leukemia (AML), differentiation, screening, plant extracts, *Adina rubella* Hance (ARH) stem, mitochondrial reactive oxygen species (mtROS), Picroside III

## Abstract

Acute myeloid leukemia (AML) is characterized by the accumulation of immature myeloid cells and a differentiation block, highlighting the urgent need for novel differentiation-inducing therapies. This study evaluated *Adina rubella* Hance (ARH) stem as a potent differentiation inducer by systematically screening 200 plant extracts. ARH stem promoted phenotypic differentiation in AML cells. In addition to its differentiation-inducing effects, ARH stem exhibited strong antileukemic activities, such as inhibiting cell proliferation, inducing cell death, and enhancing mitochondrial reactive oxygen species (mtROS) levels, the latter of which is critical for its differentiation-promoting activity. Comparative analysis with the extracts from other parts of the plant confirmed the superior efficacy of the stem extract because of its unique chemical composition. Ultra-high-performance liquid chromatography combined with quadrupole time-of-flight mass spectrometry analysis identified Picroside III as a major active compound within the stem extract, capable of recapitulating ARH stem-induced differentiation and demonstrating significant antileukemic properties. These findings underscore the therapeutic potential of ARH stem and its active component, Picroside III, as promising agents for differentiation-based treatment strategies in AML.

## 1. Introduction

The average age at diagnosis of acute myeloid leukemia (AML) is approximately 67 years, making it a typical disease of the elderly [1]. Despite the advances in diagnosis and chemotherapy, the five-year survival rate is only approximately 30% and drops to below 10% for patients over 65 years [2]. AML results from cancerous mutations in myeloid progenitor cells in the blood or bone marrow, causing abnormal cell proliferation. This disrupts blood production and weakens the immune system, leading to severe infections. Without treatment, 90% of patients die within one year, posing significant healthcare burdens as populations age rapidly [3].

AML is a heterogeneous group of disorders rather than a single disease, characterized by diverse cytogenetic and molecular features, including chromosomal abnormalities [4]. This heterogeneity leads to significant variations in the treatment response, remission rates, relapse risk, and survival outcomes among individual patients, complicating treatment efforts [5,6]. In addition, it contributes to differences in clinical prognosis, such as cure rates and the likelihood of relapse, presenting challenges in finding standardized treatment strategies tailored to the profile of each patient [5].

Remission induction therapy with chemotherapy leads to complete remission (CR) in more than 70% of AML patients under 60 years but only approximately 40% for older patients [4]. After remission, post-remission therapy is required to prevent relapse and achieve a cure. The 7 + 3 regimen (seven days of cytarabine with three days of anthracycline) remains standard but has produced limited improvements in outcomes over the last 40 years [5]. High-risk patients face a 75% relapse rate [6]. Moreover, older patients often cannot undergo intensive chemotherapy or stem cell transplantation [7], highlighting the need for new therapeutic options.

Since 2017, several new therapies for AML have been approved, showing improved outcomes. The key developments include the following: (i) Midostaurin, an FLT3 inhibitor, marked the start of the new era [8], followed by gilteritinib for FLT3-ITD mutations [9]; (ii) Ivosidenib and enasidenib target IDH mutations, which is observed in 5–15% of AML cases [10]; (iii) Venetoclax, a BCL-2 inhibitor, is approved for elderly patients over 75 years, in combination with standard treatments [11]; (iv) Gemtuzumab ozogamicin, targeting CD33, is approved for relapsed or refractory AML [12]. Nevertheless, these treatments are limited to specific patient groups, highlighting the urgent need for therapies with broader applications.

AML differentiation therapy promotes the terminal differentiation of leukemia cells, halting their proliferation and eventually triggering apoptosis. One of the most successful examples of differentiation therapy is using all-trans retinoic acid (ATRA) for acute promyelocytic leukemia (APL). APL is driven by the t(15;17) translocation, producing the PML-RARA fusion gene [13]. Standard chemotherapy, such as the 7 + 3 regimen, had limited success, with early mortality rates of 30–40% because of severe bleeding [14]. In contrast, ATRA minimizes side effects and achieves complete remission rates of over 90%, proving the effectiveness of differentiation therapy [15]. This success highlights the potential of developing similar therapies for other AML subtypes.

Plants have long served as a valuable source of medicinal compounds for treating various ailments, including inflammation, infections, and cancers. Curcumin from turmeric, Boswellia acids from frankincense, gingerol from ginger, and resveratrol from grapes exhibit potent anti-inflammatory effect [16]. In addition, some of the compounds are used widely for cancer treatment: (i) paclitaxel, derived from the Pacific yew tree, targets microtubules and is effective against various cancers, including breast cancers [17]; (ii) vincristine, sourced from the periwinkle plant, also targets microtubules to induce growth arrest and is primarily used for the treatment of lymphomas and leukemias [17]; (iii) camptothecin, extracted from the *Camptotheca acuminata* tree, inhibits topoisomerase I and is used to treat ovarian and colon cancers [18].

Plants belonging to the *Adina* genus have anti-inflammatory and anti-microbial properties. They also have been used in various folk remedies, especially for treating fevers, digestive issues, and skin problems [19,20]. This study evaluated 100 plant extracts. Of these plants, *Adina rubella* Hance (ARH) stem extracts were most effective at inducing differentiation in AML cells. This effect involves cell cycle arrest in the G2/M phase and apoptosis across multiple AML cell lines. These findings suggest that the anti-AML activity of ARH stem is linked to increased ROS production. In particular, the stems contain most of the anticancer compounds, with Picroside III identified as a key phytochemical promoting differentiation. These results suggest that ARH stem and Picroside III may be novel treatments for AML patients.

## 2. Results

### 2.1. Adina rubella Hance Stem Emerged as the Most Potent Differentiation-Inducer in a Chemical Library Screening

**Figure 1:**
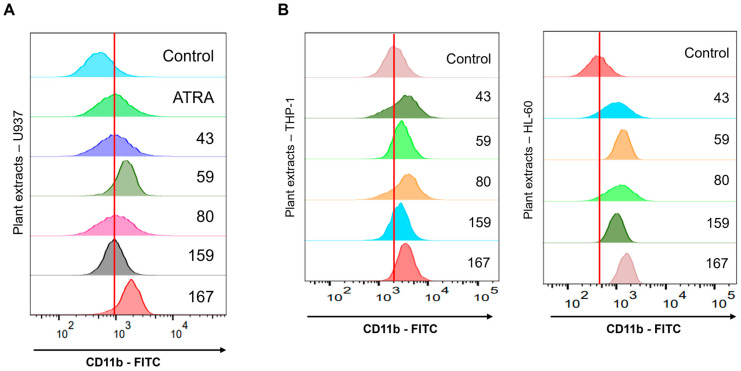
Screening test identifies promising five plant extracts of myeloid differentiation. CD11b expression was evaluated by FACS analysis following a 72-h treatment of (**A**) U937, (**B**) THP-1, or HL-60 cells with *Adina rubella* Hance stem (43), *Fumaria officinalis* L. whole plant (59), *Adonis multiflora* Nishikawa & Koki Ito root system (80), *Corydalis speciosa* Maxim. leaves (159), and *Chelidonium majus* var. *asiaticum* (H. Hara) Ohwi aerial part (167), each at a concentration of 20 µg/mL. ATRA (1 µM) was used as a positive control.

Screening was conducted on 200 plant extracts to evaluate their effects on cell differentiation using the U937 cell line and identify those with differentiation-inducing effects. None were previously assessed for their potential in treating hematologic cancers. The screening results showed that five plant extracts (*Adina rubella* Hance stem (43), *Fumaria officinalis* L. whole plant (59), *Adonis multiflora* Nishikawa & Koki Ito root system (80), *Corydalis speciosa* Maxim. leaves (159), and *Chelidonium majus* var. *asiaticum* (H. Hara) Ohwi aerial part (167)) showed more significant induction of CD11b expression compared to all-trans retinoic acid (ATRA), which promotes differentiation (Figure 1A). The ability to increase CD11b expression was assessed to determine if these five plant extracts also exhibit differentiation effects in other AML cell lines, including THP-1 and HL-60. All five plant extracts also increased CD11b expression in these additional cell lines (Figure 1B). Among the five extracts, *Fumaria officinalis* L. whole plant (59), *Corydalis speciosa* Maxim. leaves (159), and *Chelidonium majus* var. *asiaticum* (H. Hara) Ohwi aerial part (167) contain high levels of isoquinoline alkaloids [21,22,23]. Recent studies reported that these compounds induce differentiation by inhibiting LSD1 in AML cells [24]. Therefore, *Adina rubella* Hance stem (ARH stem), which exhibited the most significant increase in CD11b expression, was selected as the final candidate for further investigation, excluding these three extracts.

### 2.2. Adina rubella Hance Stem Overcomes Differentiation Block in AML Cells

**Figure 2:**
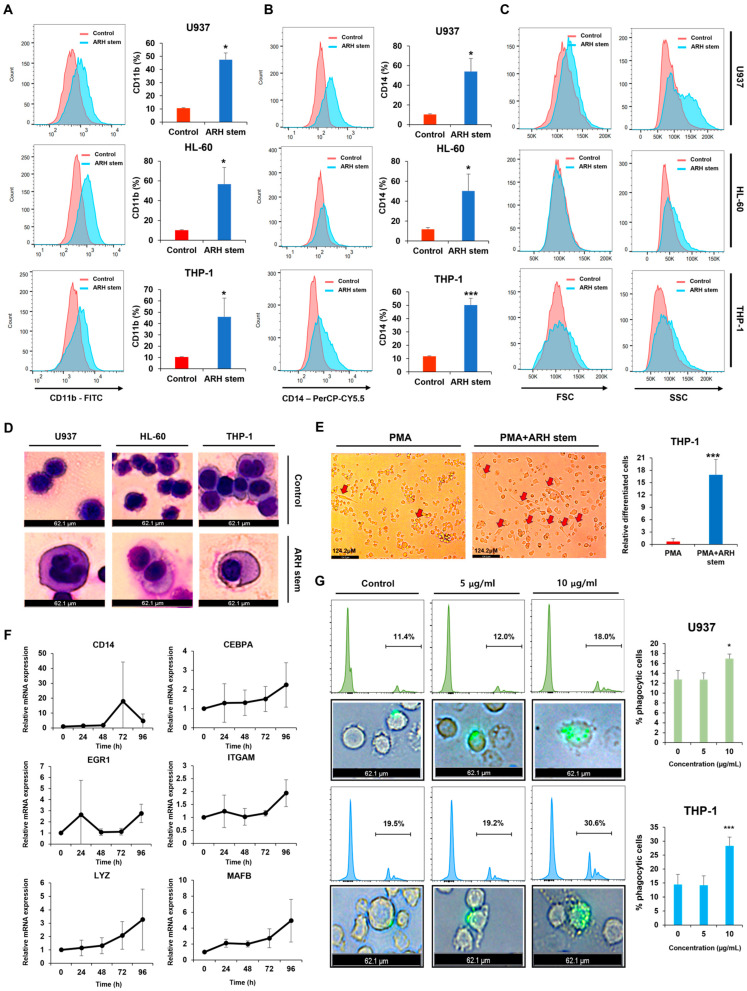
*Adina rubella* Hance stem induces phenotypic differentiation in AML cells. Human AML cell lines (U937, THP-1, and HL-60) were exposed to *Adina rubella* Hance stem extract (20 μg/mL) for 72 h. After incubation, the expression levels of CD11b (**A**) and CD14 (**B**) were analyzed using flow cytometry, and the FSC/SSC scatter profiles were assessed (**C**). (**D**) U937 and HL-60 cells were treated with *Adina rubella* Hance stem extract (20 μg/mL) for 72 h. Following incubation, Giemsa staining was performed to assess morphological changes, including an increased nuclear-to-cytoplasmic ratio and chromatin condensation. (**E**) THP-1 cells were treated with *Adina rubella* Hance stem extracts (20 µg/mL) and/or phorbol 12-myristate 13-acetate (PMA, 1 ng/mL) for five days. Morphological changes indicative of differentiation, including cell adherence and shape alterations, were observed and captured at the end of the induction period. The red arrows indicate differentiated cells. (**F**) U937 cells were treated with *Adina rubella* Hance stem extract (10 μg/mL) for 72 h, followed by qRT-PCR to measure the expression of myeloid differentiation-related genes. (**G**) U937 and THP-1 AML cells were treated with *Adina rubella* Hance stem extract (20 μg/mL) for five days. After refreshing the media, the cells were incubated with fluorescently labeled microspheres (0.5 μM) for one hour. Microsphere uptake was analyzed using flow cytometry and fluorescence microscopy. Images are representative of three independent experiments. The data are presented as mean ± SD, and statistical analysis was performed using the two-tailed Mann–Whitney U test or one-way ANOVA. * *p* ≤ 0.05; *** *p* ≤ 0.001.

The phenotypic differentiation-inducing effects of ARH stem were observed in three AML cell lines (U937, HL-60, and THP-1). ARH stem significantly upregulated CD11b levels, consistent with screening results (Figure 2A). CD14, a myeloid marker found on monocytes and macrophages, was also measured to determine if ARH stem promotes myeloid cell differentiation towards the monocyte lineage. ARH stem markedly elevated CD14 levels (Figure 2B) and induced differentiation towards the monocyte lineage. Morphological analyses validated the differentiation-inducing potential of ARH stem. FACS analysis revealed an increase in cell size (forward scatter, FSC) and intracellular complexity (side scatter, SSC), which is consistent with a differentiated cell phenotype (Figure 2C). These morphological alterations were also observed under microscopy after Giemsa staining, with an increase in cell size and a decrease in the nuclear-to-cytoplasmic ratio (Figure 2D). Because PMA treatment induces THP-1 cell differentiation into dendritic cells [25], this study investigated whether ARH stem could enhance this effect. Cells co-treated with PMA and ARH stem exhibited significantly enhanced differentiation into dendritic cells compared to PMA treatment alone (Figure 2E). The transcriptional levels of differentiation-associated genes, including *CD14*, *CEBPA*, *EGR1*, *ITGAM*, *LYZ*, and *MAFB*, were examined to assess the intracellular changes alongside these extracellular alterations. The ARH stem treatment progressively upregulated the expression of these genes (Figure 2F). Finally, ARH stem-induced monocyte-lineage cells were assessed for any phagocytic activity characteristic of normal monocytes or macrophages. FACS analysis and fluorescence microscopy showed that ARH stem treatment increased the number of phagocytic cells significantly (Figure 2G). Collectively, these findings show that ARH stem effectively promotes differentiation in AML cells.

### 2.3. Adina rubella Hance Stem Demonstrates Antileukemic Effects In Vitro

**Figure 3:**
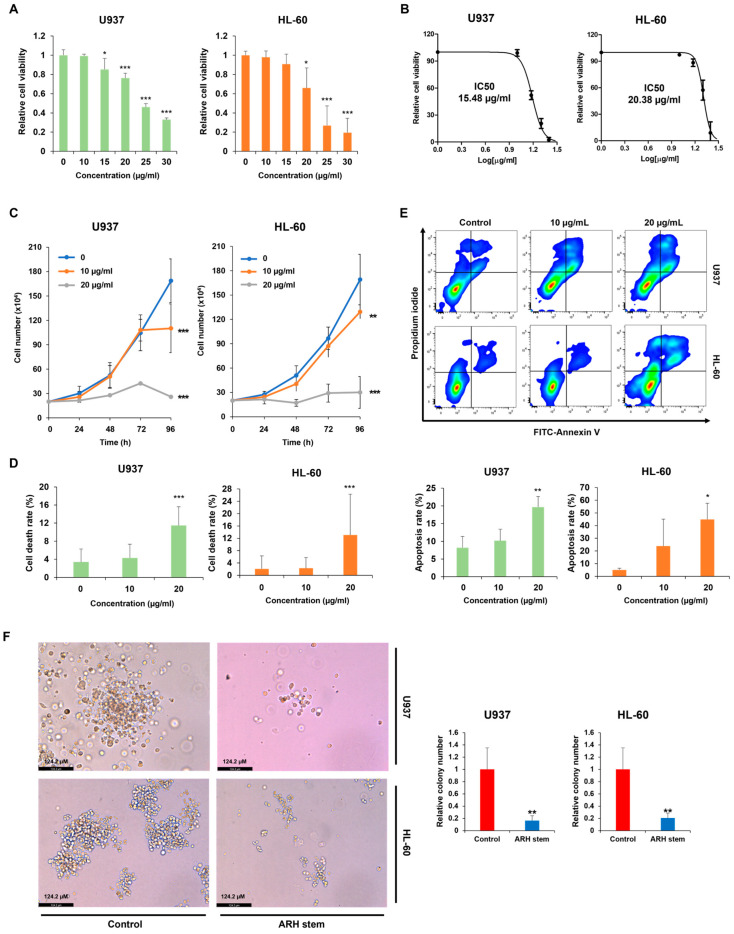
*Adina rubella* Hance stem has anti-leukemic effects. (**A**) U937 and HL-60 cells were exposed to *Adina rubella* Hance stem (0, 10, 15, 20, 25 or 30 µg/mL) for 72 h. The cell viability was measured using the MTS assays. (**B**) The IC50 values for U937 and HL-60 cells were determined using GraphPad PRISM 5 software based on the MTS assay. (**C**) U937 and HL-60 cells were exposed to *Adina rubella* Hance stem (0, 10, or 20 µg/mL) for 96 h. Cell counting was performed every 24 h to evaluate cell proliferation. (**D**) U937 and HL-60 cells were treated with *Adina rubella* Hance stem (0, 10, or 20 µg/mL) for 72 h. Cell counting was performed to evaluate the number of dead cells in U937 and HL-60 cells. (**E**) U937 and HL-60 cells were treated with *Adina rubella* Hance stem (0, 10, or 20 µg/mL) for 72 h, followed by staining with PI/Annexin V and analyzing apoptotic cells using flow cytometry. (**F**) U937 and HL60 cells were treated with *Adina rubella* Hance stem (5 µg/mL) for eight days in methylcellulose, and the colony number was observed and quantified. The images were representatives of three independent experiments. Data are represented as ± SD using the one-way ANOVA test. * *p* ≤ 0.05; ** *p* ≤ 0.01, *** *p* ≤ 0.001.

This study investigated the effects of ARH stem-induced differentiation on cell proliferation inhibition and cell death promotion. The cytotoxic effects of ARH stem were evaluated using an MTS assay, which demonstrated a dose-dependent decrease in cell viability (Figure 3A and Figure S1A). The IC50 values for U937, HL-60, and THP-1 cells were measured as 15.48, 20.48, and 11.72 µg/mL, respectively (Figure 3B and Figure S1B). Based on these values, we assessed cell proliferation and apoptosis. ARH stem inhibited cell proliferation in a dose-dependent manner (Figure 3C and Figure S1C) and induced apoptosis in AML cells (Figure 3D,E and Figure S1D,E). Additionally, a methylcellulose-based colony formation assay revealed that ARH stem reduced the number of colonies (Figure 3F). These findings underscore the antileukemic potential of ARH stem.

### 2.4. Adina rubella Hance Stem Promotes the Differentiation of AML Cells by Enhancing Mitochondrial Ros Levels and Upregulating p21 Expression

**Figure 4:**
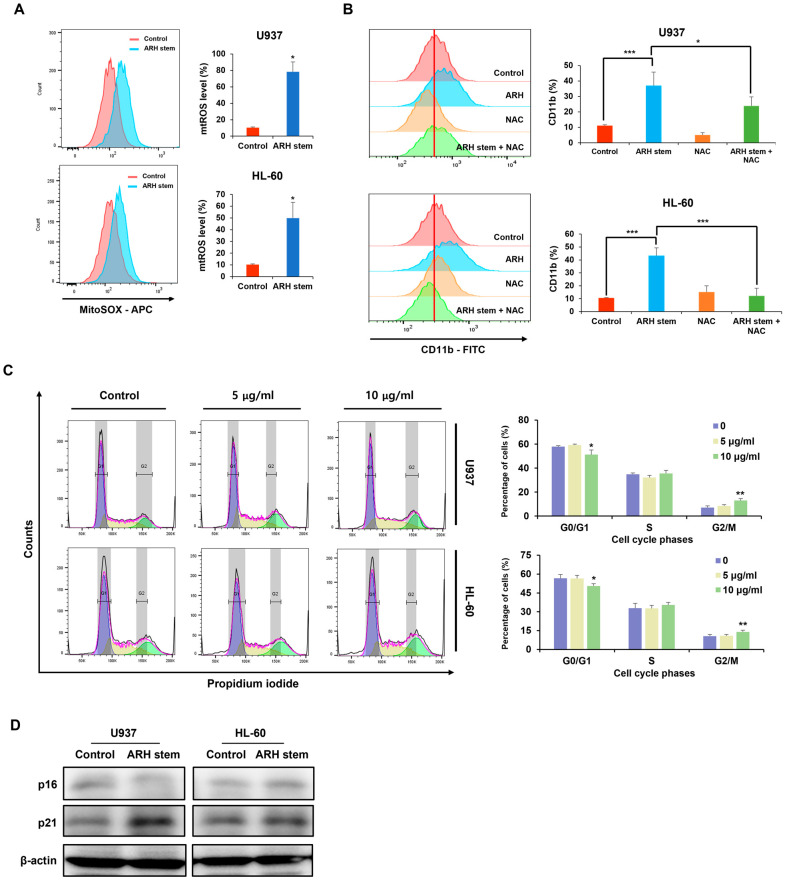
*Adina rubella* Hance stem induces AML cell differentiation via elevated mitochondrial ROS and p21 upregulation. U937 and HL-60 cells were treated with *Adina rubella* Hance stem extract (20 μg/mL) for 72 h. (**A**) Mitochondrial ROS levels were measured by flow cytometry using MitoSOX Red staining. (**B**) Cells were pre-incubated with or without 1 mM NAC (a ROS scavenger) for one hour, followed by treatment with *Adina rubella* Hance stem extract (20 μg/mL) for 72 h, and mitochondrial ROS levels were re-analyzed. (**C**) Cell cycle distribution was assessed by flow cytometry after 72-h treatment with *Adina rubella* Hance stem extract (0, 5, or 10 μg/mL). (**D**) Western blotting was performed to analyze p16 and p21 expression levels after 48-h incubation with *Adina rubella* Hance stem extract (20 μg/mL). β-actin was used as the internal control. The data are presented as mean ± SD, and statistical analysis was performed using the two-tailed Mann–Whitney U test or one-way ANOVA. * *p* ≤ 0.05; ** *p* ≤ 0.001; *** *p* ≤ 0.0001.

The mitochondrial ROS (mtROS) levels were evaluated to explore the intracellular changes triggered by the ARH stem treatment. The ARH stem treatment elevated mtROS levels significantly in AML cells (Figure 4A). AML cells were co-treated with ARH stem and the ROS scavenger NAC to determine if elevated mtROS contribute to ARH stem-induced differentiation. Flow cytometry revealed a decrease in CD11b expression when ARH stem was combined with NAC, compared to the treatment with ARH stem alone. Flow cytometry revealed a decrease in CD11b expression when ARH stem was combined with NAC compared to the ARH stem treatment alone (Figure 4B). Hence, the ARH stem-induced elevation in mtROS levels plays a key role in driving differentiation in AML cells. The accumulation of mtROS affects the cell cycle. An analysis of the cell cycle showed that the ARH stem-induced anticancer effects are linked to cell cycle regulation by inducing G2/M arrest (Figure 4C). p16 and p21 expression were assessed to determine which regulatory factors are involved, considering that U937 and HL-60 are cell lines with p53 mutations. ARH stem upregulated p21 expression in both cell lines, suggesting that ARH stem modulates p21 expression and is involved in regulating the cell cycle (Figure 4D).

### 2.5. Adina rubella Hance Stem Exhibits a Distinctive Potential as a Differentiation Inducer for Aml Cells, Distinguishing It from Other Parts of the Plant

**Figure 5:**
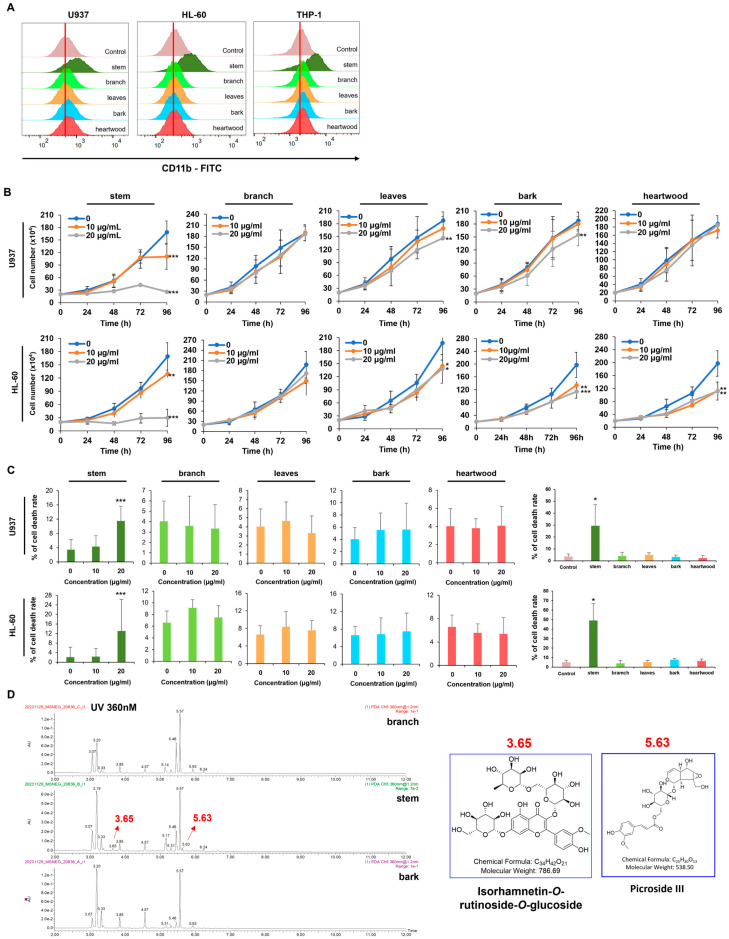
*Adina rubella* Hance stem shows the ability to be an AML differentiation inducer distinct from other parts of the plant. (**A**) U937 and HL-60 cells were treated with 20 μg/mL of extracts from the stems, branches, leaves, bark, and heartwood of *Adina rubella* Hance for 72 h. The CD11b expression levels were evaluated after treatment by flow cytometry. (**B**) U937 and HL-60 cells were exposed to *Adina rubella* Hance stem, branch, leaves, bark, and heartwood (0, 10, or 20 µg/mL) for 96 h. Cell counting was performed every 24 h to evaluate cell proliferation (**C**) U937 and HL-60 cells were exposed to *Adina rubella* Hance stem, branch, leaves, bark, heartwood (0, 10, or 20 µg/mL) for 72 h. Cell counting was performed to evaluate the number of dead cells in U937 and HL-60 cells. (**D**) The components of the branch, stem, and bark of *Adina rubella* Hance were analyzed by UPLC QTOF-MS to identify the molecules that function as differentiation inducers. These data showed that the stem extract had two distinctive peaks (3.65, 5.63). The data are represented as ±SD using the one-way ANOVA test. * *p* ≤ 0.05; ** *p* ≤ 0.01, *** *p* ≤ 0.001.

This study evaluated whether the differentiation-inducing ability is specific to its stem extract by treating AML cells with extracts from four other plant parts (branch, leaf, bark, and heartwood) at the same concentration, comparing their antileukemic effects with those of the stem extract. An analysis of CD11b expression in three AML cell lines (U937, HL-60, and THP-1) showed that only the stem extract upregulated CD11b expression. In contrast, the other extracts had little or no effect (Figure 5A). Subsequent evaluations of cell proliferation and cell death revealed distinct responses to the extracts. The leaf and bark extracts inhibited cell proliferation in U937 and HL-60 cell lines, while the heartwood extract suppressed proliferation only in HL-60 cells. Nevertheless, the cells treated with these extracts exhibited higher proliferation than those treated with the stem extract (Figure 5B). Furthermore, only the stem extract increased cell death at the same concentration (Figure 5C). These results suggest that the stem extract contained unique differentiation-inducing components absent in the other extracts or had a higher concentration of these components. Comparative UPLC-QTOF-MS analysis of the branch, stem, and bark extracts identified two unique peaks (3.65 and 5.63) in the stem extract, which were tentatively identified as isorhamnetin-O-rutinoside-O-glucoside (3.65) and Picroside III (5.63) (Figure 5D).

### 2.6. Picroside III Exhibits Potential as a Therapeutic Agent for Inducing Differentiation in U937 Cells

**Figure 6:**
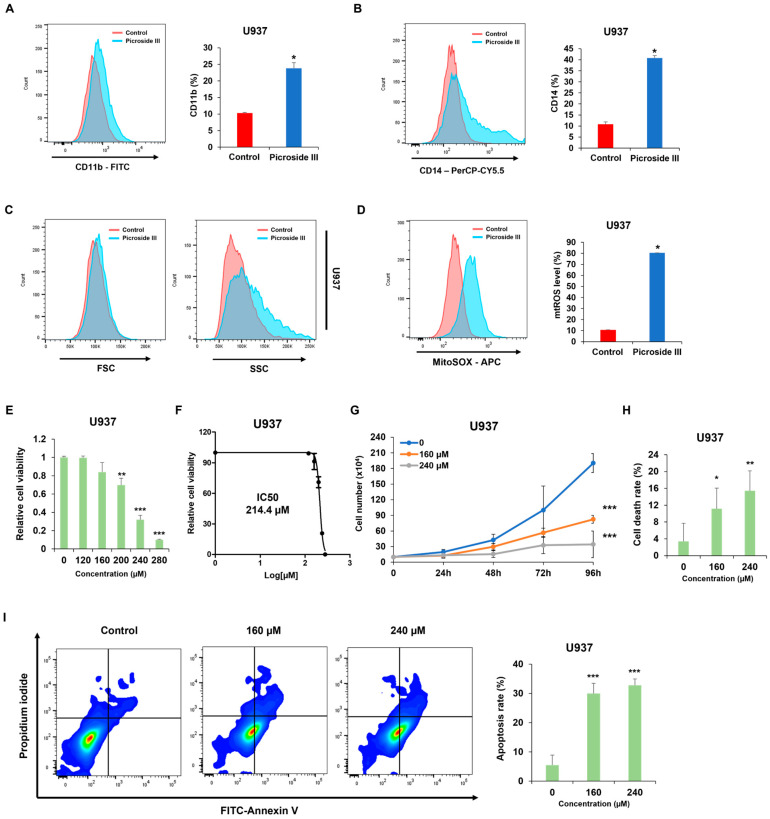
Picroside III induces differentiation and demonstrates antileukemic activity in U937 cells. U937 cells were exposed to Picroside III (160 μM) for 72 h, after which the CD11b (**A**), CD14 (**B**), and FSC/SSC (**C**) ratios were analyzed using flow cytometry. (**D**) Mitochondrial ROS levels were assessed by flow cytometry using MitoSOX Red staining after treatment with Picroside III (160 μM) for 72 h. (**E**) U937 cells were exposed to Picroside III (0, 120, 160, 200, 240, or 280 μM) for 72 h. The cell viability was measured using the MTS assays. (**F**) The IC50 values for U937 cells were determined using GraphPad PRISM 5 software based on the MTS assay results. (**G**) Cell proliferation was measured every 24 h for 96 h after treatment with Picroside III (0, 160, or 240 μM). (**H**) Cell death was quantified after treatment with Picroside III (0, 160, or 240 μM) for 72 h. (**I**) U937 cells were treated with Picroside III (0, 160, or 240 μM) for 72 h, followed by staining with PI/Annexin V and analyzing apoptotic cells using flow cytometry The data are presented as mean ± SD and statistical analysis was performed using the two-tailed Mann–Whitney U test or one-way ANOVA. * *p* ≤ 0.05; ** *p* ≤ 0.001, *** *p* ≤ 0.001.

FACS analysis validated the effects of two molecules predicted in the previous analysis on CD11b expression. Among the two molecules, only Picroside III significantly upregulated CD11b expression (Figure 6A). Further measurements of CD14 expression showed that Picroside III promotes differentiation into a monocyte lineage by markedly increasing CD14 expression (Figure 6B). FSC and SSC parameters were utilized to characterize the morphological changes in U937 cell differentiation induced by Picroside III (Figure 6C). Additionally, Picroside III elevated the mtROS levels, similar to the effects of ARH stem (Figure 6D). An MTS assay was performed to confirm whether Picroside III, like ARH stem, possesses not only differentiation effects but also antileukemic activity. The results indicated that Picroside III also caused toxicity in U937 cells (Figure 6E). Using the IC50 values (214.4 μM) obtained (Figure 6F), we evaluated cell proliferation and apoptosis. Picroside III inhibited cell proliferation in a dose-dependent manner (Figure 6G) and induced apoptosis in U937 cells (Figure 6H,I). These findings demonstrate that Picroside III possesses differentiation potential and cytotoxicity in U937 cells.

## 3. Discussion

This study reports the findings of a screening involving 200 plant extracts to identify novel differentiation-inducing agents for the treatment of acute myeloid leukemia (AML). Natural products often possess unique mechanisms that inhibit cancer cell proliferation or induce differentiation, making them invaluable resources for developing innovative therapies. For instance, Sea buckthorn, Dog Rose, Garden Sage, and Oregano extracts have been shown to inhibit AML cell growth and promote differentiation when combined with vitamin D [26]. Similarly, Jungermannenone C produces reactive oxygen species in AML cells, driving differentiation and suggesting its therapeutic potential [27]. Building on these precedents, this study undertook an extensive screening of understudied plant extracts and identified *Adina rubella* Hance (ARH) stem extract as a potent differentiation-inducing agent for AML cells.

Previous studies have shown that *Adina rubella* Hance extracts have biological effects, including antioxidant, anti-inflammatory, and anticancer activities. For example, the *Adina rubella* Hance extract inhibited nitric oxide (NO) production via activated macrophages. In addition, the extracts from the leaves of *Adina rubella* Hance and grandifloroside derived from these leaves had antioxidant and anti-inflammatory effects. Furthermore, the root extract of the *Adina rubella* Hance extract exhibited anticancer activity in vitro against the human colon cancer cell line LS174T. On the other hand, the antileukemic potential of the stem extract of *Adina rubella* Hance has not been evaluated. The mechanism by which ARH-induced ROS upregulates p21 and p16 CKI expression, leading to cell cycle arrest and differentiation in AML cells, remains unclear. Previous research suggests that the DNA damage response (DDR) pathway and p38 MAPK are crucial in regulating the levels of p21 and p16, respectively [28,29,30]. ROS produced by ARH may activate p38 through phosphorylation, which in turn could enhance p16 transcription. Meanwhile, p21 expression might be elevated when ARH-induced ROS causes double-strand breaks (DSBs), triggering DDR signaling. One key effector of DDR is the p53 tumor suppressor, which activates p21 transcription. Intriguingly, however, p53 is functionally deficient in U937 and HL60 AML cells, indicative of a potential p53-independent mechanism. Notably, one study demonstrated that NF-kappa B can increase p21 levels by directly binding to its promoter in p53-deficient HL60 cells [31], suggesting that NF-kappa B may be activated by DDR signaling in U937 and HL60 cells. Further research is needed to clarify the roles of ROS/p16 and ROS/DDR/NF-kappa B/p21 pathways in AML cells (Figure S2). This study is the first to show that the stem extract of *Adina rubella* Hance had antileukemic effects.

UPLC-QTOF-MS analysis identified two unique peaks in ARH, predicted to be Isorhamnetin-O-rutinoside-O-glucoside and Picroside III. Functional evaluation of these compounds’ anti-leukemic effects demonstrated that only Picroside III was able to replicate the differentiation-inducing activity of ARH. Picroside III is a glycoside, a compound consisting of a sugar molecule attached to a non-sugar molecule, typically a flavonoid or terpenoid. It is primarily found in the root of the *Picrorhiza kurroa* plant, which grows in the Himalayan region. While Picroside III is known for its anti-inflammatory effects in the colon [32,33], its potential role in AML and other hematologic malignancies remains uncharted. The findings of this study indicate that Picroside III may influence differentiation in AML cells; however, further research is necessary to establish its therapeutic potential for hematologic cancers. It is also important to consider whether the observed effects stem from Picroside III alone or a synergistic action of multiple compounds present in the extract.

These findings suggest that the CDK inhibitor p21 is upregulated in response to ARH stem exposure, suggesting its involvement in ARH stem-induced AML differentiation. Based on previous reports, p21 may play multifaceted roles in this process. For example, C/EBPα interacts with p21 to inhibit CDK2 activity, promoting growth arrest. This effect is lost when the interaction between C/EBPα and p21 is disrupted, even if the interaction of C/EBPα with DNA remains intact. ARH stem exposure enhanced C/EBPα and p21 expression. Therefore, this study hypothesized that their interaction contributes to AML differentiation and growth arrest. Previous research showed that p21 directly regulates SOX2 expression, a critical factor in neural progenitor cell expansion. Accordingly, ARH stem-induced p21 upregulation may influence the transcription of genes related to AML differentiation. Nevertheless, the precise mechanisms through which p21 regulates AML differentiation remain to be elucidated.

While this study establishes ARH and Picroside III as promising differentiation-inducing agents for AML, several challenges remain. ARH and Picroside III have not been evaluated for their efficacy and safety in animal models, which is critical for assessing their clinical potential. Moreover, their combinatorial effects with existing AML treatments, such as cytarabine or doxorubicin, have not been tested, leaving open the possibility of synergistic therapeutic benefits. Additionally, further research into optimizing the pharmacokinetic properties of Picroside III, including its bioavailability and drug delivery efficiency, is essential. Despite these limitations, this study provides a foundation for developing ARH and Picroside III as novel therapeutic strategies, offering new insights into their mechanisms of action and potential clinical applications for AML treatment.

## 4. Materials and Methods

### 4.1. Reagents

Plant extracts used in this research were obtained from The Korea Plant Extract Bank at the Korea Research Institute of Bioscience and Biotechnology (Daejeon, Korea). *Adina rubella* Hance stem (KPM046-013), branch (KPM046-012), leaves (KPM008-079), bark (KPM009-087), heartwood (KPM009-086) were deposited in The Korea Plant Extract Bank at the Korea Research Institute of Bioscience and Biotechnology (Daejeon, Korea). Picroside III (purity > 99.52%) was purchased from Medchemexpress (Monmouth Junction, NJ, USA; HY-N0409), and its purity was confirmed using liquid chromatography-mass spectrometry (LC-MS). The following primary antibodies were used for western blots: p16 (1:1000 dilution, Cell Signaling Technology (CST), Danvers, MA, USA; 80772S), p21 (1:1000 dilution, Cell Signaling Technology (CST), Danvers, MA, USA; 2947S), and β-actin (1:5000 dilution, Santa Cruz Biotechnology, Dallas, TX, USA; sc-4778). HRP-conjugated anti-rabbit and anti-mouse secondary antibodies were obtained from Bethyl Laboratories, Inc. (A120-101p and A90-116p-33; Montgomery, TX, USA). N-acetylcysteine amide (NAC) was purchased from Sigma (Sigma, St. Louis, MO, USA; A9165).

### 4.2. AML Cell Lines and Cell Culture

The human AML cell lines (U937, THP-1, and HL-60) were purchased from the Korean Cell Line Bank (Seoul, Korea). The cells were maintained in RPMI-1640 medium (Hyclone, Logan, UT, USA) supplemented with 10% fetal bovine serum (FBS; Hyclone, Melbourne, VIC, Australia), 1% L-glutamine, 1% N-2-hydroxyethylpiperazine-N′-2-ethanesulfonic acid (HEPES) buffer, and 1% penicillin/streptomycin. The cells were cultured in a humidified atmosphere containing 5% CO_2_ at 37 °C. The control group was treated with the same medium containing DMSO at a final concentration of 0.2% (*v*/*v*) to match the solvent conditions of the treated groups. This ensures that any observed effects in the experimental groups are not due to the presence of DMSO but rather the specific treatments applied.

### 4.3. Analysis of Phenotypic Differentiation by Flow Cytometry

Phenotypic differentiation was evaluated by treating AML cells with *Adina rubella* Hance stem, branch, leaves, bark, heartwood extracts (20 µg/mL), and Picroside III (160 µM) in 12-well plates for 72 h. After treatment, the cells were stained for one hour at 4 °C with FITC anti-mouse/human CD11b antibody (1:100 dilution, BioLegend, San Diego, CA, USA; catalog number 101206) or PerCP-Cy™5.5 Mouse Anti-Human CD14 antibody (1:100 dilution, BD Biosciences, Franklin Lakes, NJ, USA; catalog number 550787). Based on previously established methods [34], flow cytometry was conducted using a BD FACSAria™ Fusion Flow Cytometer (BD Biosciences, Becton Drive, Franklin Lakes, NJ, USA). Data were then analyzed using FlowJo version 7.6 software (TreeStar).

### 4.4. Measurement of Cell Viability and IC50 Value

Cell viability was measured using the CellTiter 96 AQueous One Solution [3-(4,5-dimethylthiazol-2-yl)-5-(3-carboxymethoxyphenyl)-2-(4-sulfophenyl)-2H-tetrazolium] cell proliferation assay (MTS, Promega, Madison, WI, USA). U937, HL-60, and THP-1 cells were treated with *Adina rubella Hance* stem extracts at concentrations of 0, 10, 15, 20, 25, or 30 µg/mL for 72 h. U937 cells were treated with Picroside III at concentrations of 0, 120, 160, 200, 240, or 280 µM for 72 h. The IC50 values were determined using the GraphPad PRISM 5 software based on the MTS assay results.

### 4.5. Giemsa Staining

Giemsa staining was conducted using previously established protocols [35]. AML cells were incubated with *Adina rubella* Hance stem extracts (20 µg/mL) at a density of 2.0 × 10^5^ cells per well in 12-well plates for three days. Subsequently, the cells were fixed with 100% methanol for five minutes, air-dried, and stained with Giemsa solution (Sigma, 48900-500ML-F, St. Louis, MO, USA) for 15 min. The cellular morphology was examined by optical microscopy (LEICA DMi8, Leica Microsystems, Wetzlar, Germany) at 400× magnification. Representative images were obtained using Leica Application Suite X (LAS X) software (Leica Microsystems, Wetzlar, Germany).

### 4.6. Real-Time Quantitative RT-PCR (qRT-PCR)

The expression levels of genes associated with AML cell differentiation, including CD14, CEBPA, EGR1, ITGAM, LYZ, and MAFB, were evaluated using real-time quantitative RT-PCR (qRT-PCR). According to the manufacturer’s protocol, total RNA was extracted from cells using the TRIzol reagent (Thermo Fisher Scientific, Waltham, MA, USA). cDNA was synthesized using the PrimeScript RT Reagent Kit (Takara, RR047A, Kusatsu-shi, Japan) in a reaction mixture containing total RNA, random hexamers, and oligo(dT) primers. The synthesis reaction was performed under the following thermal conditions: 37 °C for 15 min (reverse transcription) and 85 °C for 5 s (enzyme inactivation). qRT-PCR was conducted using the TOPreal qPCR PreMIX SYBR Green with low ROX (Enzynomics, RT500M, Daejeon, Republic of Korea) in a QuantStudio 5 Real-Time PCR System (Applied Biosystems, Waltham, MA, USA). Reactions were performed in a total volume of 20 µL, containing 3 µL of synthesized cDNA, 10 µL of SYBR Green, 2 µL of each forward and reverse primer (10 µM), and nuclease-free water to adjust the final volume. The qRT-PCR thermal cycling program consisted of initial denaturation at 95 °C for 10 min, followed by 40 cycles of denaturation at 95 °C for 10 s, annealing at 59 °C for 30 s, and extension at 72 °C for 30 s. A melting curve analysis was conducted from 59 °C to 95 °C to confirm the specificity of the amplified products. TBP (TATA-box binding protein) was used as the internal control for normalization of target gene expression levels. The relative expression of each gene was calculated using the 2^−ΔΔCt^ method, and primer sequences used in the analysis are provided in Supplementary Table S1.

### 4.7. Phagocytosis Assays

A phagocytosis assay was conducted using reported methodologies [36,37]. AML cells were treated with *Adina rubella* Hance stem extracts in six-well plates for 120 h. The media were then refreshed, and the cells were cultured for another 24 h. Phagocytosis was measured by incubating the cells with fluorescent microspheres (F8823, Invitrogen, Carlsbad, CA) for one hour. The phagocytic cells were analyzed with a BD FACSAria™ Fusion Flow Cytometer (BD Biosciences, Becton Drive, Franklin Lakes, NJ, USA) and visualized under a LEICA DMi8 fluorescence microscope (Leica Microsystems, Wetzlar, Germany) at 200× magnification. Leica Application Suite X (LAS X) software (Leica Microsystems, Wetzlar, Germany) captured representative images, and FlowJo 7.6 software (TreeStar) was used for data analysis.

### 4.8. Induced Differentiation of THP 1 Cells

The induced differentiation assay of THP-1 cells was conducted based on previously established protocols [25] with slight modifications. THP-1 cells were seeded at a density of 3.0 × 10^5^ cells per well in 6-well plates. The cells were treated with *Adina rubella* Hance stem extracts (20 µg/mL) and/or phorbol 12-myristate 13-acetate (PMA, 1 ng/mL) for five days under standard culture conditions (37 °C, 5% CO_2_, and a humidified atmosphere). For the experimental conditions, PMA alone served as the control group, and the combination of PMA and *Adina rubella* Hance stem extracts was used as the treatment group. At the end of the induction period, the morphological changes associated with differentiation, such as adherence and changes in cell shape, were observed and imaged. The cells were visualized under a LEICA DMi8 inverted microscope (Leica Microsystems, Wetzlar, Germany) at 400× magnification. Multiple fields were selected randomly to ensure representative imaging of the entire well. Images were captured using the integrated camera system and analyzed using Leica Application Suite X (LAS X) software (Leica Microsystems, Wetzlar, Germany) for quantitative and qualitative assessment. Differentiation markers were evaluated by observing characteristic changes in morphology, such as flattening and adherence of cells to the plate surface, indicative of macrophage-like differentiation. All experiments were performed in triplicate to ensure reproducibility, and control conditions (PMA treatment alone) were used to compare the differentiation effects of the treatments.

### 4.9. Cell Proliferation Assays

A trypan blue dye exclusion assay was conducted to assess the effects of *Adina rubella* Hance stem, branch, leaf, bark, heartwood extracts, and Picroside III on the proliferation of AML cells. The AML cells were treated with these extracts in 12-well plates. Trypan blue (Sigma, St Louis, MO, USA) staining was performed daily for four consecutive days. The concentration of trypan blue used was 0.4%, and cells were stained by mixing an equal volume of 0.4% trypan blue solution with the cell suspension at a 1:1 ratio. The stained cells were then analyzed after 1 min. The viable cell count was then determined using a hemocytometer.

### 4.10. Apoptosis Assays

Annexin V/PI staining was performed to evaluate the apoptosis rates of AML cells. AML cells were exposed to *Adina rubella* Hance stem extracts (10 or 20 µg/mL) in a 24-well plate for 72 h. U937 cells were treated with Picroside III at concentrations of 0, 160, or 240 µM in a 24-well plate for 72 h. The apoptosis rate was then analyzed by flow cytometry (BD FACSAria™ Fusion Flow Cytometer, BD Biosciences, Becton Drive Franklin Lakes, NJ, USA) after staining with an Annexin V-FITC/PI (fluorescein isothiocyanate/propidium iodide) apoptosis detection kit (Cat# 556547, BD Biosciences, San Jose, CA, USA).

### 4.11. Cell Cycle Analysis

The cell cycle distribution of AML cells was assessed as described elsewhere [34]. Briefly, AML cells were cultured in a 12-well plate at a density of 2 × 10^5^ cells per well with *Adina rubella* Hance stem extracts for 72 h under standard culture conditions (37 °C, 5% CO_2_, and humidified atmosphere). Following incubation, the cells were harvested by gentle pipetting to ensure minimal cell loss, washed twice with 1X PBS (phosphate-buffered saline, pH 7.4) to remove any residual culture medium or extract, and subsequently fixed in 75% ethanol. The fixation was performed by slowly adding pre-chilled ethanol dropwise while gently vortexing the cell suspension to prevent clumping. The cells were then incubated at 4 °C for 40 min to allow proper permeabilization and preservation of cellular structures. After fixation, the cells were centrifuged at 2000 rpm for 5 min at room temperature (21 °C), and the ethanol was carefully aspirated. The pellet was resuspended in 1 mL of a PI staining solution, which consisted of 40 µg/mL propidium iodide (PI), 0.1% Triton X-100, and 100 µg/mL RNase A prepared in PBS. The samples were incubated in the dark at 37 °C for 1 h to ensure complete staining of DNA and degradation of RNA, which could otherwise interfere with the analysis. Cell cycle analysis was conducted using flow cytometry (BD FACSAria™ Fusion Flow Cytometer, BD Biosciences, Franklin Lakes, NJ, USA), and the data were analyzed with FlowJo 7.6 software (TreeStar). Doublets and debris were excluded based on forward and side scatter profiles. The resulting data were further analyzed using FlowJo 7.6 software (TreeStar), with cells gated into sub-G1, G1, S, and G2/M phases based on their DNA content.

### 4.12. Colony Formation Assays

The colony-forming ability of AML cells was assessed using colony-formation assays, as described elsewhere [34]. U937 cells were plated at a density of 1.0 × 10^4^ cells per well, and HL-60 cells were plated at 5.0 × 10^3^ cells per well in 12-well plates containing methylcellulose (MethoCult™ H4100; STEMCELL Technologies, Vancouver, BC, Canada; 04100) with *Adina rubella* Hance stem extracts. The cells were incubated for eight days in a humidified atmosphere containing 5% CO_2_ at 37 °C. After incubation, colony formation was evaluated microscopically. The colonies were imaged using a LEICA DMi8 (Leica Microsystems, Wetzlar, Germany) at 400× magnification, and representative images were analyzed using Leica Application Suite X (LAS X) software (Leica Microsystems, Wetzlar, Germany). Images were captured from three distinct areas of each well to count cell colonies. Colonies consisting of at least approximately 50 cells were manually counted by visual inspection, and the average number of colonies from these areas was used for quantification.

### 4.13. Western Blots

AML cells were cultured with *Adina rubella* Hance stem extracts (20 µg/mL) for 24 or 48 h and lysed in Igepal CA-630 NP-40 buffer (Sigma, St. Louis, MO, USA; 9002-93-1), supplemented with 1 mM sodium vanadate, 50 mM β-glycerophosphate disodium salt, 142 mM β-mercaptoethanol (BioWORLD, Irving, TX, USA), ProteaseArrest™ (G-Biosciences, St. Louis, MO, USA), and 5 mM EDTA (G-Biosciences). The samples were heated to 100 °C for 10 min in a sample buffer, separated by polyacrylamide gel electrophoresis, and transferred to Immobilon-P Transfer membranes for two hours. The membranes were blocked with 1% bovine serum albumin (BSA; MP Biomedicals, Santa Ana, CA, USA) and incubated overnight with the primary antibodies at 4 °C. After three washes with Tris-buffered saline containing 0.1% Tween 20 (TBS-T) for five minutes each, the membranes were incubated with anti-mouse/rabbit secondary antibodies for one hour at room temperature. After another round of washing (three times for 10 min each), chemiluminescent detection was performed using EzWestLumi Plus (ATTO, Tokyo, Japan). The protein bands were visualized with a Luminograph II (ATTO).

### 4.14. Measurement of Mitochondrial ROS Levels

The mitochondrial ROS levels were measured by treating the AML cells with *Adina rubella* Hance stem (20 µg/mL), and Picroside III (160 µM) for 72 h, followed by incubating the relevant cells with 5 μM MitoSox Red (Medchemexpress, NJ, USA; HY-D1055) at 37 °C for 10 min. Flow cytometry was performed after washing the cells twice with PBS.

### 4.15. UPLC QTOF-MS Analysis

Ultra-high-performance liquid chromatography (UPLC) and quadrupole time-of-flight mass spectrometry (QTOF-MS) were used for quantitative phytochemical analysis. An automated XEVO-QTOF-MS mass spectrometer with an electrospray ionization (ESI) source and an ACQUITY UPLC I-Class system (Waters Corporation, Milford, MA, USA) was used for analysis using UPLC-QTOF-MS. Various settings were used, including a source temperature of 110 °C, capillary voltage of 2.3 kV, and cone voltage of 40 V. The desolvation gas flow rate was set to 800 L/h, with a desolvation temperature of 350 °C. The scan duration was set to 0.2 s with an acquisition mass range of 100–1500 m/z in both positive (ESI⁺) and negative (ESI⁻) ion modes. Chromatographic analysis was conducted using a 100 mm-long ACQUITY UPLC^®^ BEH C18 column with a 2.1 mm internal diameter (2.1 × 100 mm, 1.7 μm, Waters Corporation, Milford, MA, USA). The samples were separated using the following gradient program: water containing 0.1% formic acid as mobile phase A and acetonitrile containing 0.1% formic acid as mobile phase B; column temperature set to 35 °C; the flow rate was maintained at 0.4 mL/min. The samples were prepared at 3000 ppm in methanol, and 1 µL of each sample was injected into the UPLC system. A photodiode array (PDA) detector monitored the absorbance at 254 nm with a spectral range of 190–500 nm and a sampling rate of 10 points/s.

### 4.16. Statistical Analysis

All experiments were repeated at least three times independently. GraphPad PRISM 5 software (GraphPad Software Inc., San Diego, CA, USA) was used for statistical analyses. A Mann–Whitney U test or one-way ANOVA followed by Tukey’s post hoc analysis was conducted to compare the significance.

## 5. Conclusions

*Adina rubella* Hance (ARH) stem exhibited differentiation-inducing properties in AML cells, along with potent antileukemic effects, including inhibition of cell proliferation, induction of cell death, and enhancement of mitochondrial reactive oxygen species (mtROS) levels. Picroside III, suggested as the active component of the ARH stem extract, demonstrated differentiation-inducing potential. These findings highlight the therapeutic potential of ARH stem and Picroside III as promising agents for differentiation-based treatment strategies in AML.

## Data Availability

The original contributions presented in this study are included in the article/Supplementary Materials. Further inquiries can be directed to the corresponding author.

## Supplementary Materials

The following supporting information can be downloaded at https://www.mdpi.com/article/10.3390/ijms26031350/s1.
