# Supplementary material for: Therapeutic Potential of Adina rubella Hance Stem and Picroside III as a Differentiation Inducer in AML Cells via Mitochondrial ROS Accumulation
Source: Int J Mol Sci. 2025 Feb 5;26(3):1350. doi: 10.3390/ijms26031350 (PMC11818474; doi:10.3390/ijms26031350)
Supplement: Supplementary file 1 [file ijms-26-01350-s001.zip › Supplementary figure.pptx]

## Slide 1
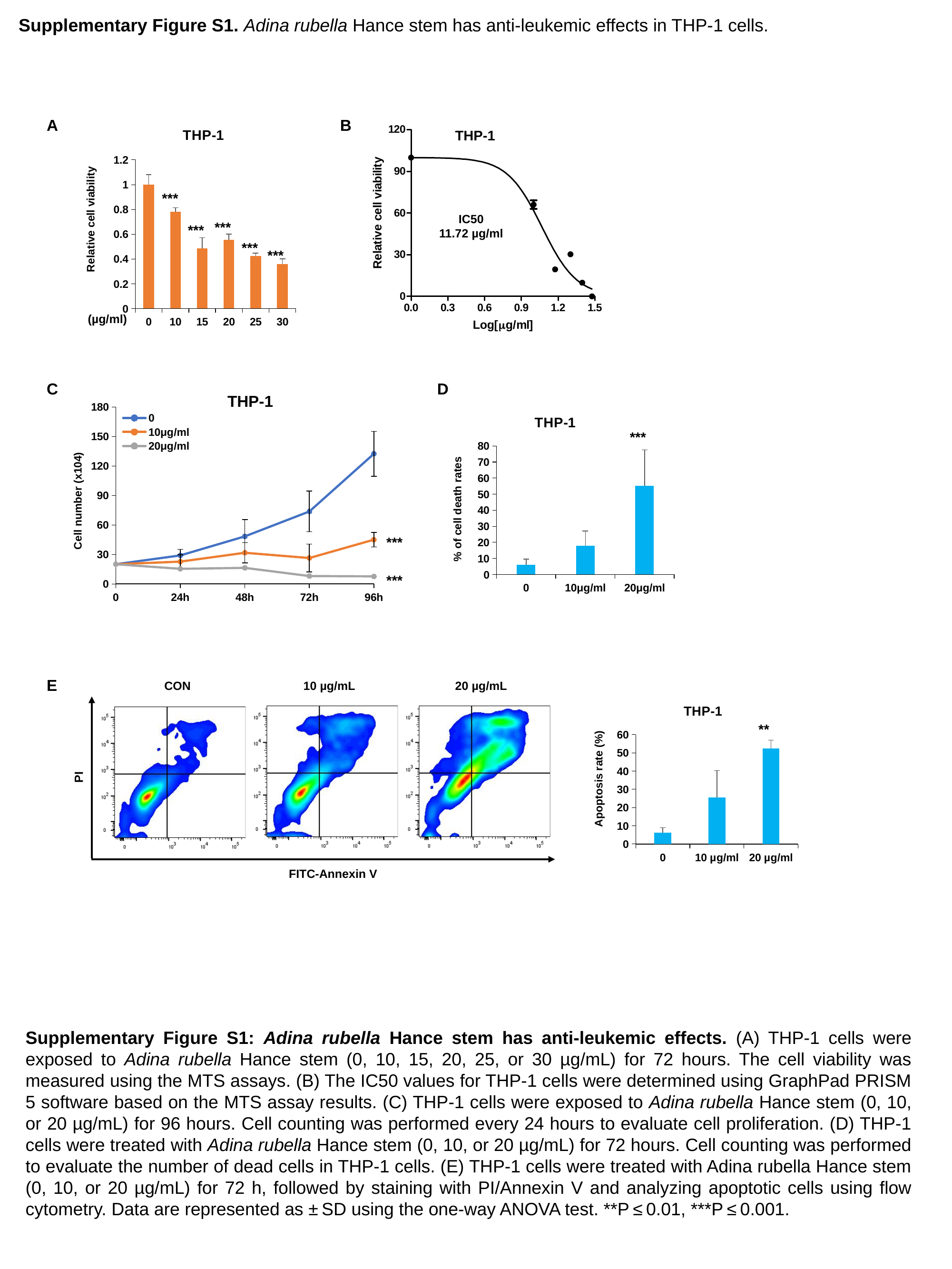

Supplementary Figure S1. Adina rubella Hance stem has anti-leukemic effects in THP-1 cells.
A
B
### Chart: THP-1
| Category | ARS |
|---|---|
| 0 | 1.0 |
| 10 | 0.7824562247159477 |
| 15 | 0.48394039254398696 |
| 20 | 0.5535706163018022 |
| 25 | 0.42240018848111804 |
| 30 | 0.3590650586493235 |THP-1
***
IC50
11.72 µg/ml
***
***
***
***
(µg/ml)
C
D
THP-1
### Chart
| Category | 0 | 10μg/ml | 20μg/ml |
|---|---|---|---|
| 0 | 20.0 | 20.0 | 20.0 |
| 24h | 29.0 | 22.666666666666668 | 15.333333333333334 |
| 48h | 48.333333333333336 | 31.666666666666668 | 16.333333333333332 |
| 72h | 73.66666666666667 | 26.333333333333332 | 8.0 |
| 96h | 132.33333333333334 | 45.0 | 7.666666666666667 |
### Chart: THP-1
| Category | 72h |
|---|---|
| 0 | 5.958264108670613 |
| 10μg/ml | 17.778213166144198 |
| 20μg/ml | 55.33068783068783 |***
***
***
E
CON
10 µg/mL
20 µg/mL
### Chart: THP-1
| Category | apoptosis |
|---|---|
| 0 | 6.333333333333333 |
| 10 µg/ml | 25.44666666666667 |
| 20 µg/ml | 52.343333333333334 |
**
PI
FITC-Annexin V
Supplementary Figure S1: Adina rubella Hance stem has anti-leukemic effects. (A) THP-1 cells were exposed to Adina rubella Hance stem (0, 10, 15, 20, 25, or 30 µg/mL) for 72 hours. The cell viability was measured using the MTS assays. (B) The IC50 values for THP-1 cells were determined using GraphPad PRISM 5 software based on the MTS assay results. (C) THP-1 cells were exposed to Adina rubella Hance stem (0, 10, or 20 µg/mL) for 96 hours. Cell counting was performed every 24 hours to evaluate cell proliferation. (D) THP-1 cells were treated with Adina rubella Hance stem (0, 10, or 20 µg/mL) for 72 hours. Cell counting was performed to evaluate the number of dead cells in THP-1 cells. (E) THP-1 cells were treated with Adina rubella Hance stem (0, 10, or 20 µg/mL) for 72 h, followed by staining with PI/Annexin V and analyzing apoptotic cells using flow cytometry. Data are represented as ± SD using the one-way ANOVA test. **P ≤ 0.01, ***P ≤ 0.001.

## Slide 2
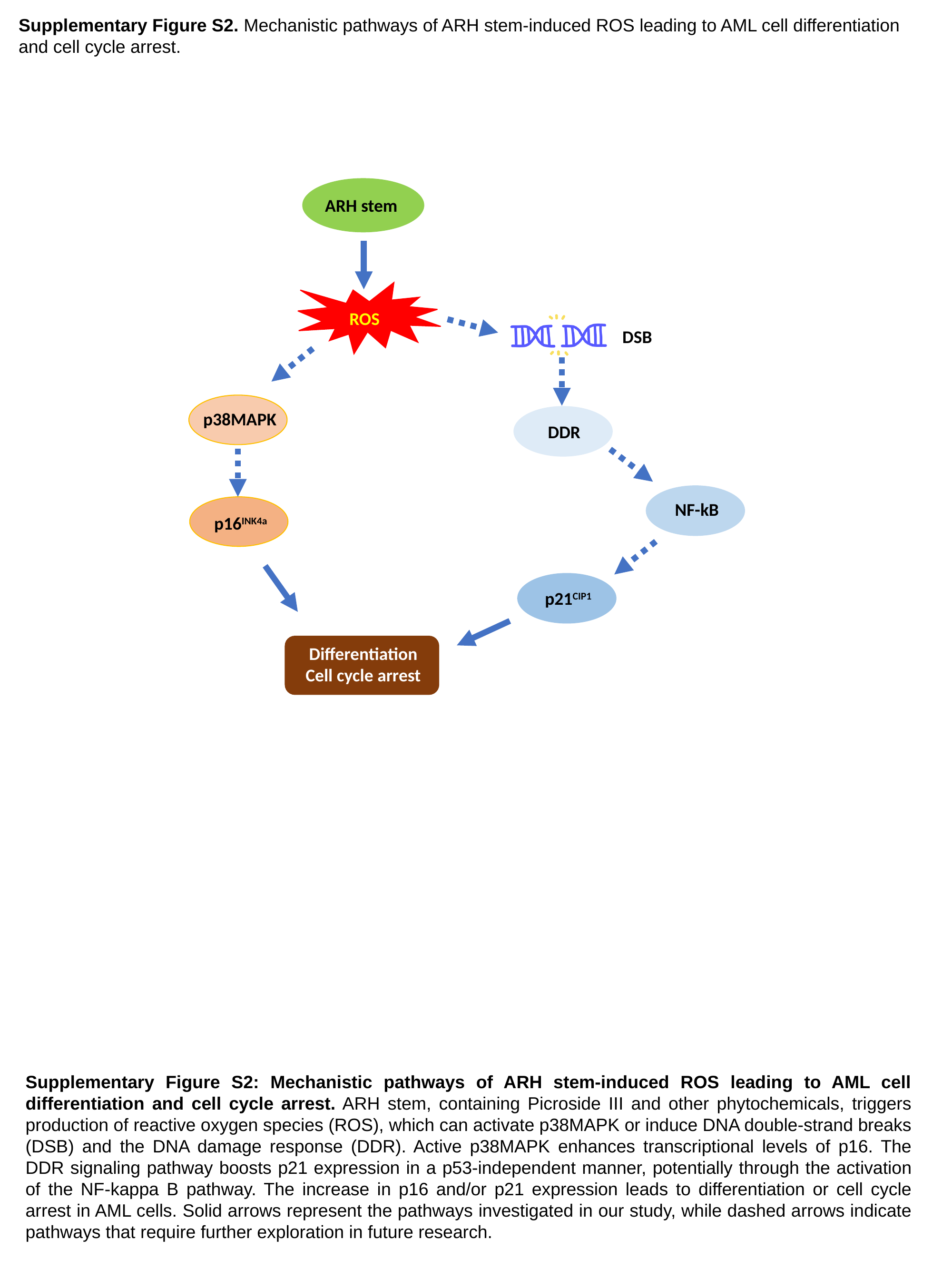

Supplementary Figure S2. Mechanistic pathways of ARH stem-induced ROS leading to AML cell differentiation and cell cycle arrest.
ARH stem
ROS
DSB
p38MAPK
DDR
NF-kB
p16INK4a
p21CIP1
Differentiation
Cell cycle arrest
Supplementary Figure S2: Mechanistic pathways of ARH stem-induced ROS leading to AML cell differentiation and cell cycle arrest. ARH stem, containing Picroside III and other phytochemicals, triggers production of reactive oxygen species (ROS), which can activate p38MAPK or induce DNA double-strand breaks (DSB) and the DNA damage response (DDR). Active p38MAPK enhances transcriptional levels of p16. The DDR signaling pathway boosts p21 expression in a p53-independent manner, potentially through the activation of the NF-kappa B pathway. The increase in p16 and/or p21 expression leads to differentiation or cell cycle arrest in AML cells. Solid arrows represent the pathways investigated in our study, while dashed arrows indicate pathways that require further exploration in future research.
