# Supplementary material for: Therapeutic Potential of Adina rubella Hance Stem and Picroside III as a Differentiation Inducer in AML Cells via Mitochondrial ROS Accumulation
Source: Int J Mol Sci. 2025 Feb 5;26(3):1350. doi: 10.3390/ijms26031350 (PMC11818474; doi:10.3390/ijms26031350)
Supplement: Supplementary file 1 [file ijms-26-01350-s001.zip › Supplementary Table.pdf]

Supplementary Table S1. List of primers used for the qPCR analysis

Primer ID	Forward	Reverse
TBP	TATAATCCCAAGCGGTTTGCTGCG	AATTGTTGGTGGGTGAGCACAAGG
ITGAM	AGAACAACATGCCCAGAACC	GCGGTCCCATATGACAGTCT
CD14	CTGCAACTTCTCCGAACCTC	CCAGTAGCTGAGCAGGAACC
CEBPA	AACCTTGTGCCTTGGAAATG	CCCTATGTTTCCACCCCTTT
EGR1	TGACCGCAGAGTCTTTTCCT	TGGGTTGGTCATGCTCACTA
LYZ	GCCAAATGGGAGAGTGGTTA	ATCACGGACAACCCTCTTTG
MAFB	GCCTGCGCTAATTGTAGGAG	CGCACTTGAAAGTTGCAAAA
